# NPC1 promotes HTNV replication by controlling innate immune response

**DOI:** 10.3389/fimmu.2026.1811629

**Published:** 2026-06-05

**Authors:** Hansong Qi, Yuechun Wu, Rong Wei, Shengyao Chen, Jumin Zhou, Wei Hou, Xinglou Yang

**Affiliations:** 1 Key Laboratory of Genetic Evolution and Animal Models, Yunnan International Joint Laboratory of Zoonotic Viruses, Yunnan Key Laboratory of Biodiversity Information, Kunming Institute of Zoology, Chinese Academy of Sciences, Kunming, China; 2 Kunming College of Life Science, University of Chinese Academy of Sciences, Kunming, China; 3 State Key Laboratory of Virology, Institute of Medical Virology, School of Basic Medical Sciences, Wuhan University, Wuhan, China

**Keywords:** glycoprotein, HTNV, innate immunity, NPC1, protein interaction

## Abstract

Hantaan virus (HTNV) causes the majority of hemorrhagic fever with renal syndrome (HFRS) across Asia, which imposes a substantial public health burden. Virus–host interaction, particularly protein–protein interactions, essentially determined the clinic outcomes of HFRS patients; however, the interactions between HTNV and host proteins remain largely uncharacterized. To identify host factors interacting with the HTNV glycoprotein (GP), we combined deep-learning-based virtual screening via MaSIF with immunoprecipitation coupled to mass spectrometry (IP-MS) and found that host cholesterol transporter protein Niemann-Pick C1 (NPC1) binds to HTNV GP and promotes HTNV replication. Specifically, NPC1 mitigates anti-viral innate immune response following HTNV infection, a function not previously documented among its known roles in viral infection. Our findings not only expand current understandings of NPC1 involving viral infection but also highlight its potential as an anti-viral target.

## Introduction

Hantaan virus (HTNV), the major causative agent of hemorrhagic fever with renal syndrome (HFRS), is a severe rodent-borne zoonosis that continues to cause substantial morbidity and mortality across Asia (1–3). Despite extensive surveillance and vaccination efforts engaged in endemic regions, more than 20,000 people suffered from HFRS annually, and the global burden of HFRS remains a significant public health concern (3–8). Clinical outcomes of HFRS patients are the result of an intricate interplay between HTNV and host, which usually provides us with potential therapeutic targets. Of note, protein–protein interaction between viral proteins and host proteins bridged viral–host interactions from the molecular scale to the individual level. However, the HTNV–host interaction network remains to be described, and the engaged host factors in HTNV infection are also largely unidentified, which is important to gain insight into viral life cycle and confers a promising therapeutic target for hantavirus infection. Importantly, how to rapidly map virus–host interactions is a question not only faced by HTNV research but also prevalent throughout the broader field of virology. The emerging deep-learning-driven methods hopefully enable us to map and identify the viral–host protein interaction with much lower economic and time costs. Specifically, AlphaFold, ESMFold, Boltz, and RoseTTAFold series were the most popular methods for predicting protein complex structures (9–16), which were successfully applied in identifying functional protein–protein interactions across multiple research areas (17–23). In addition, the MaSIF series recently emerged as powerful toolkits to handle tasks including protein–protein and protein–ligand screening and designing (24–26). The MaSIF-search and MaSIF-seed modules demonstrate good performance in protein–protein interaction prediction (24–26). Although the effectiveness of deep-learning-based methods in protein–protein interaction identification has been proven, the virtual screen should be combined with traditional wet lab methods to potently identify viral–host protein interactions.

NPC1, an endosomal cholesterol transporter, participates in multiple steps of the viral life cycle in numerous viruses (27). Involvement in the viral entry process is the most well-studied function of NPC1, including Ebola virus (EBOV) (28–30), Marburg viruses (31), severe acute respiratory syndrome coronavirus 2 (32), Chikungunya virus (33), dengue virus (34), hepatitis A virus (35), hepatitis E virus (36), African swine fever virus (37), and pseudorabies virus (38). In detail, NPC1 directly binds to EBOV GP to mediate viral envelope and late endosomal membrane fusion (28, 30); this is a common mechanism by which NPC1 promotes viral entry. Additionally, NPC1 could maintain cellular cholesterol homeostasis to ensure sufficient cholesterol in the plasma membrane, which is critical to clathrin-mediated endocytosis (38). Besides viral entry, NPC1 is also involved in various steps of the viral life cycle, such as viral replication factor formation (hepatitis C virus, HCV) (39, 40), viral particle release (reoviruses, HCV) (41, 42), collaborated with viral proteins (human immunodeficiency virus, hepatitis B virus) (43–45), and in some uncharacterized steps (yellow fever virus, type I feline coronavirus) (46, 47). The diverse functions of NPC1 during viral infections underscore its potential as an antiviral target, and the engagement of NPC1 in HTNV life cycle remains to be elucidated.

To gain insight into HTNV–host interaction landscapes, we combined the MaSIF virtual screen with immunoprecipitation coupled with mass spectrometry (IP-MS) and found that NPC1 interacted with HTNV GP as well as promoted viral infection. In detail, NPC1 regulates cellular cholesterol distributions to mitigate anti-viral innate immune response following HTNV infection but not the engagement of viral entry process. NPC1 attenuating innate immune response to benefit HTNV replication has been never characterized before, which broadened our knowledge on the functions of NPC1 involved in viral infection. Our findings provide additional evidence positioning NPC1 as a promising antiviral target.

## Results

### MaSIF screen combined with IP-MS identified the interaction between HTNV GP and NPC1

**Figure 1:**
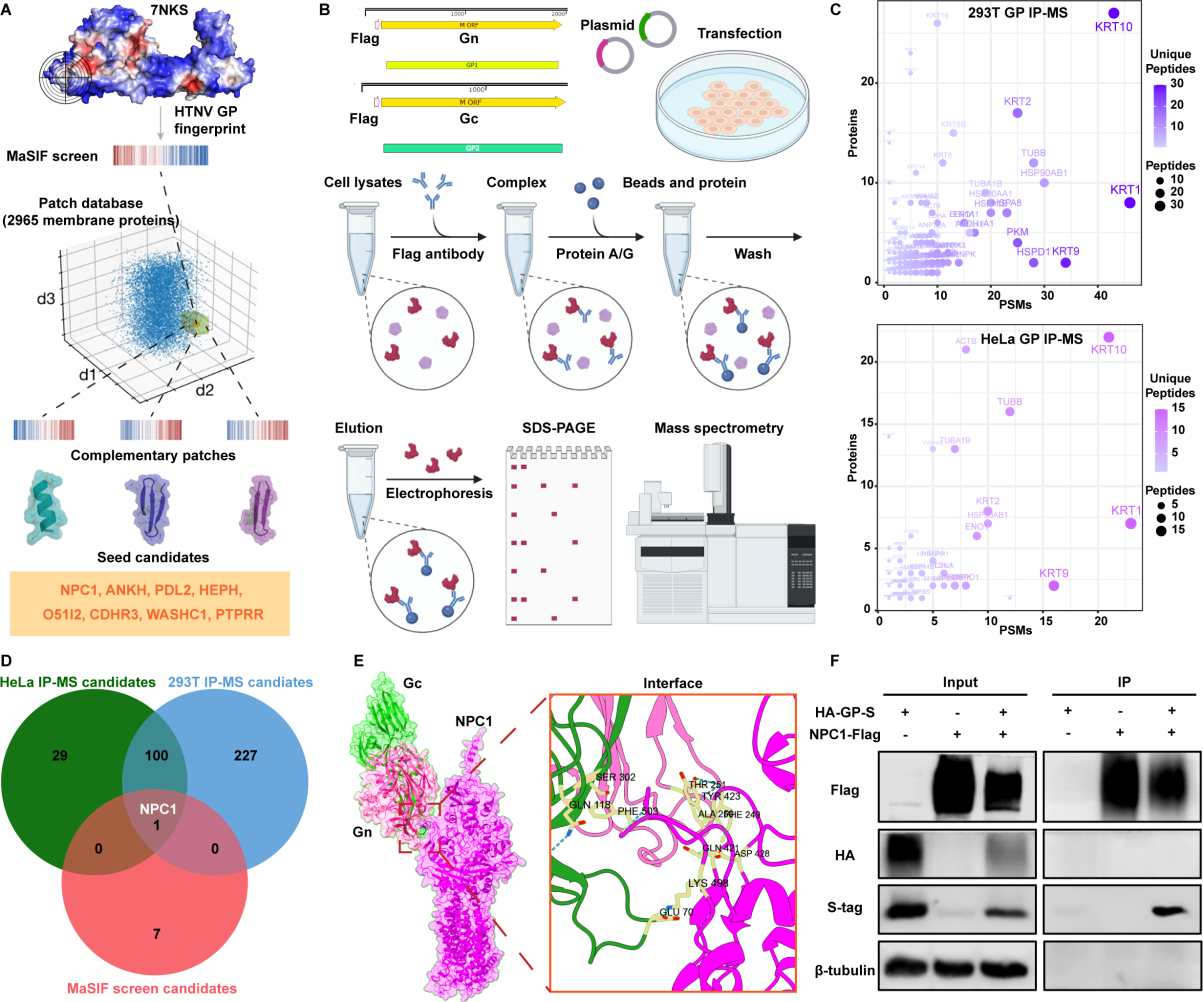
NPC1 interacts with HTNV GP. **(A)** The diagram of the MaSIF-seed screening workflow is cited from the original paper (24, 26), and the outputted candidates are listed in the orange box (for the detailed screening steps, see “Materials and methods”). **(B)** Experiment design of the HTNV GP IP-MS assay. **(C)** Enriched candidates in 293T and HeLa cells shown as scatter plot based on the IP-MS assay described in **(B)**. PSMs, the matching score between the MS raw data to peptide database; peptides, matched peptide number of the candidate protein; unique peptides, matched peptide unique to the candidate protein; proteins, the number of proteins in their protein group. **(D)** Overlapped candidates between IP-MS results and MaSIF screening results shown as a Venn diagram. **(E)** Visualization of HTNV GP–NPC1 complex outputted by AlphaFold3 with the binding interface view zoomed in. **(F)** The lysates of 293T cells overexpressed with Flag tagged NPC1 and HA and S-tag tagged HTNV GP were conveyed to perform Flag-specific immunoprecipitation, and the elution content was detected using Flag, HA, S-tag, and β-tubulin antibodies through western blot.

Protein–protein interaction usually confers biological significance. HTNV GP, a multi-functional viral protein, plays a vital role in the viral entry process. To identify the host factors which interact with HTNV GP, especially entry factors, we employed a deep-learning-driven virtual screen approach to obtain initial candidates using the MaSIF toolkits. Following the standard screen flow, we firstly extract the surface fingerprint of GP based on HTNV GP crystal structure 7NKS (48); 2,965 human membrane proteins subsequently served as the input candidates for the matching program to be performed, and eight proteins were finally predicted to interact with HTNV GP, including NPC1, ANKH, PDL2, HEPH, O51I2, CDHR3, WASHC1, and PTPRR (**Figure 1A**). Those factors were docked with GP protein by Alphafold3 (**Supplementary Figure 1A**), and the interaction interfaces between the candidates and GP were all located to the extracted interaction hot spot area in the MaSIF flow (**Figure 1A**; **Supplementary Figure 1A**), which supported the effectivity of MaSIF screening. Of note, the N-terminal flexible domain of GP, where NPC1 and ANKH were coincidentally predicted to bind to, was exposed on the surface of the HTNV viral particle, which enabled NPC1 and ANKH to be potential entry factors (**Supplementary Figure 1A**).

To comprehensively profile the engaged host factors, HTNV GP immunoprecipitation coupled with mass spectrometry (IP-MS) was performed (**Figure 1B**). 293T and HeLa cells were transfected with HTNV-GP-expressing plasmid, and the consequent IP-MS results showed that KRT and HSP protein were mostly enriched (**Figure 1C**), and the immunoprecipitated proteins from HeLa cells were greatly overlapped with that enriched from 293T cells (**Figures 1C, D**). Importantly, when intersecting the results derived from MaSIF with those obtained from IP-MS, NPC1 emerged as the only common gene (**Figure 1D**). We consequently got insight into the predicted GP–NPC1 complex; the short flexible domain located in the MLD domain of NPC1, where NPC2 and EBOV GP physically bound to NPC1 (49), interacted with HTNV GP (**Figure 1E**). Specifically, a hydrogen bond formed between the 70th glutamate of HTNV GP and the 498th lysine of NPC1 in the interface (**Figure 1E**), which might have driven this interaction. Finally, the GP–NPC1 interaction was confirmed using a Flag-specific Co-IP assay between ectopically expressed NPC1-Flag and HTNV GP (HA and S-tag tagged) in 293T cells (**Figure 1F**).

Taken together, we identified an interaction between NPC1 and HTNV GP that may play a role in the viral life cycle by combining MaSIF-based virtual screening with IP-MS. However, whether this interaction is a direct physical interaction and the corresponding binding interface remain to be experimentally validated.

### NPC1 promotes HTNV replication

**Figure 2:**
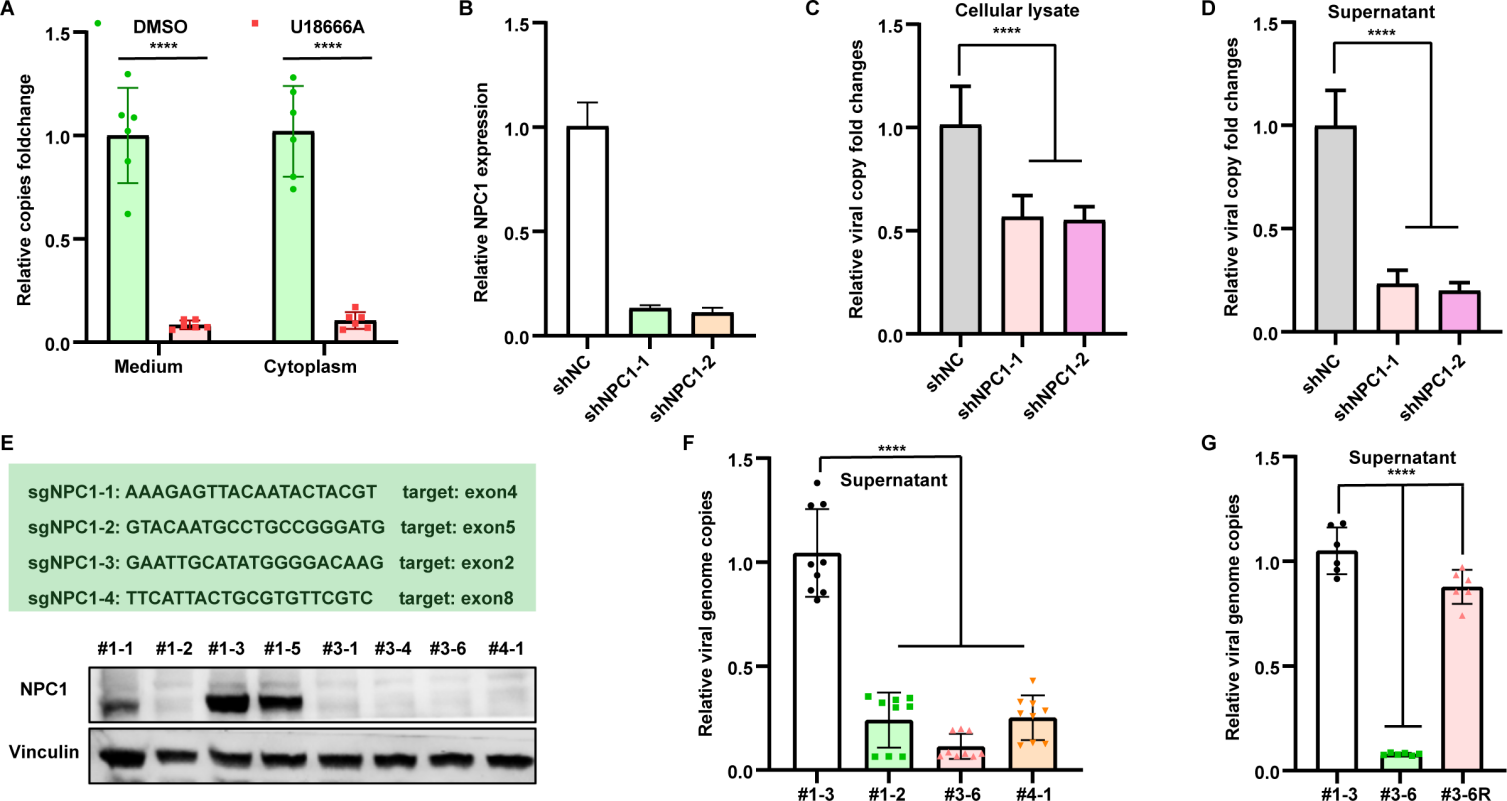
NPC1 promotes HTNV replication. **(A)** HeLa cells were infected with HTNV at MOI = 1 following pre-treatment with 10 µM U18666A for 12 h, and the viral genome copies within medium and cells were measured using qPCR at 48 h post-infection. **(B)** The relative expression level of NPC1 was quantified in both wild-type HeLa cells and shRNA-transduced HeLa cells. **(C, D)** Wild-type and NPC1 knockdown HeLa cells were infected with HTNV at MOI = 1, and the viral genome copies of cellular **(C)** and medium **(D)** were quantified using qPCR. **(E)** NPC1 protein levels of each NPC1 knockout cell clone were detected by NPC1 antibody, and vinculin was the loading control. **(F)** NPC1 knockout cells were infected with HTNV (MOI = 1) for 48 h, and the viral copies in medium were quantified using qPCR. All clone IDs are corresponding to panel (**E**). Clones #1–3 underwent clone formation process similar with others and failed to knockout NPC1, so we defined #1–3 as the control group. **(G)** NPC1 knockout failed control (#1–3), NPC1 knockout (#3–6), and NPC1 rescued (#3-6R) HeLa cells were infected with HTNV (MOI = 1) for 48 h, and the viral copies in medium were quantified using qPCR. Statistic significance: *, p<0.05; **, p<0.01; ***, p<0.001; ****, p<0.0001; ns, not significant.

To test the roles of NPC1 in HTNV infection, we pretreated HeLa cells with cholesterol transport inhibitor U18666A for 12 h followed by HTNV infection, which could mimic NPC1 knockout context. The U18666A treatment statistically repressed HTNV replication (**Figure 2A**), which is further supported by impeded HTNV replication in NPC1 knockdown cells (**Figures 2B–D**). Those results indicated that NPC1 was engaged in viral life cycle to favor HTNV replication. To further prove the observed effects of NPC1 in HTNV infection, NPC1 knockout was achieved using CRISPR-Cas9 gene editing system. Among the four designed targets, only #2 sgRNA failed to knockout NPC1 (**Figure 2E**). We therefore chose the knockout clone from the remaining three targets to re-evaluate the function of NPC1 in viral infection. Distinct sgRNA targets generated NPC1 knockout cells that consistently depressed HTNV replication (**Figure 2F**), while rescuing NPC1 expression from #3–6 cell clone greatly restored the HTNV replication in NPC1 knockout cells (**Figure 2G**). These results potently proved the favorable roles of NPC1 in HTNV life cycle. In summary, NPC1 could facilitate HTNV replication through NPC1–GP interaction.

### NPC1 is not involved in HTNV entry process

**Figure 3:**
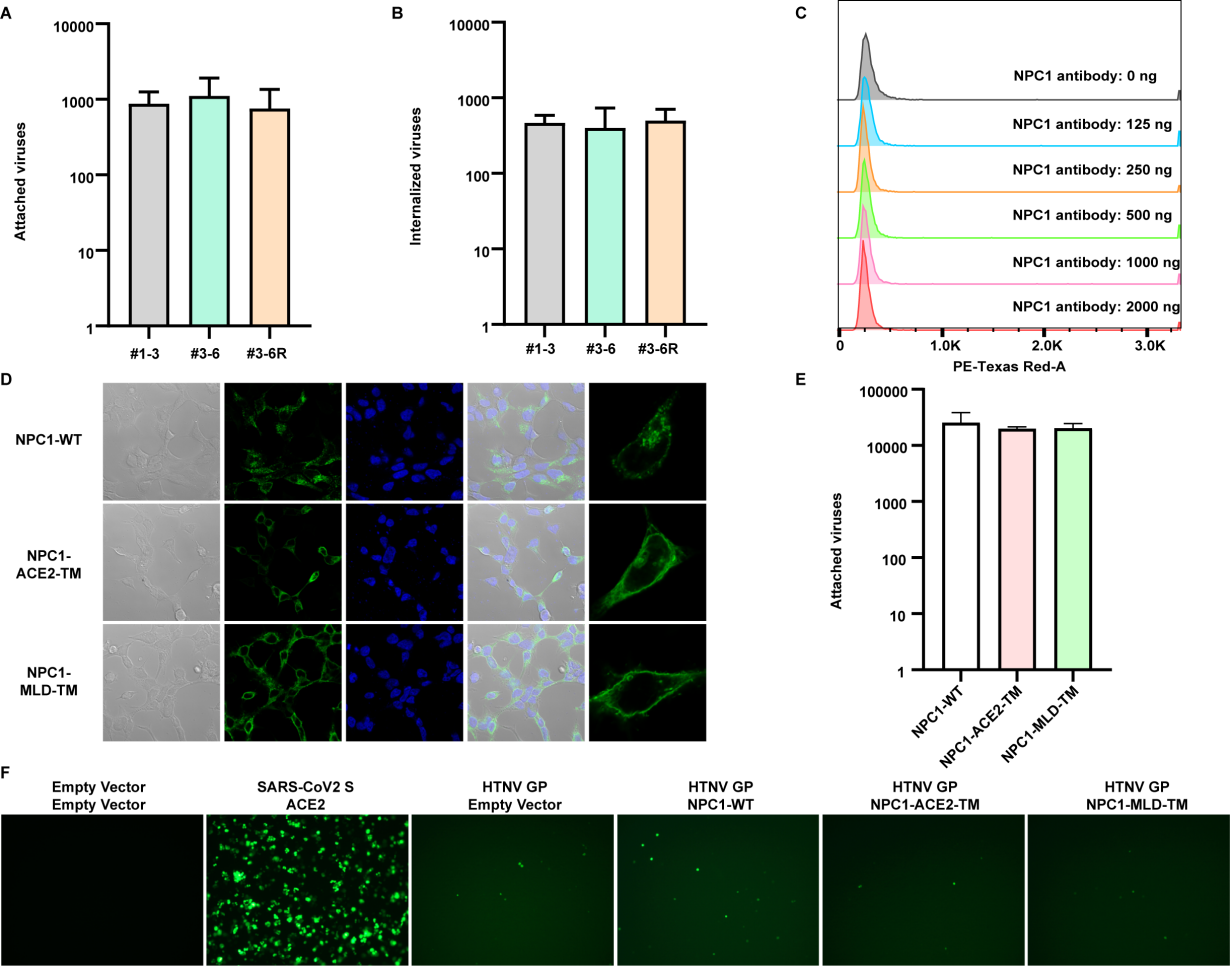
NPC1 is not implicated in the HTNV entry process. **(A, B)** NPC1 knockout failed control (#1–3), NPC1 knockout (#3–6), and NPC1 rescued (#3-6R) HeLa cells were inoculated with HTNV (MOI = 10) at 4°C for 2 h, then the harvested sample was defined as viral attachment **(A)**, and the left parallel cells were replaced to 37°C for 2 h, which was defined as viral penetration **(B)**. Viral genomes were extracted and quantified using qPCR (for the detailed steps, see “Materials and methods”). **(C)** NPC1 antibody was incubated with HeLa cells at indicated concentrations for 1 h, then HTNV was inoculated into the cells at 4°C for 2 h, and the cells were fixed for viral staining by immunofluorescence assay with NP antibody. HTNV binding to cell membrane was quantified via flow cytometry. **(D)** 293T cells were transfected with NPC1 constructs (NPC1-WT, NPC1-ACE2-TM, and NPC1-MLD-TM) for 24 h, and then protein localization was visualized by immunofluorescence staining by Flag antibody. **(E)** 293T cells were transfected with NPC1 constructs (NPC1-WT, NPC1-ACE2-TM, and NPC1-MLD-TM) for 24 h, and then the cells were inoculated with HTNV (MOI = 10) at 4°C for 2 h. Viral genome copies of the harvested sample were quantified using qPCR. **(F)** NPC1 constructs, GFP1–10 and HTNV GP, 16×GFP11 overexpressed 293T cells were co-cultured for 24 h, and the membrane fusion efficiency was quantified by the GFP signal captured by the microscope. ACE2, GFP1–10 and SARS-CoV2 S, 16×GFP11 overexpressed 293T cells were the positive control (for the detailed steps, see “Materials and methods”).

NPC1, determined as the host spectrum of EBOV infection, is a well-documented entry factor of EBOV and other viruses (27, 50, 51). Due to the determinative roles of NPC1 in EBOV entry and the interaction interface between NPC1 and HTNV GP overlapping with that occupied by EBOV GP, we speculated that NPC1 might also be involved in the HTNV entry process. However, we did not observe any statistically significant difference in HTNV attachment and penetration processes (**Figures 3A, B**). Furthermore, NPC1 antibody incubation also showed a dispensable effect in blocking HTNV attached to the cell surface (**Figure 3C**). NPC1 has been reported to localize mainly in the late endosomal membrane, and such subcellular localization was also shown in HeLa cells (**Figure 3D**). Although NPC1 did not participate in the cell-membrane-related viral entry process, we cannot exclude the possibility that NPC1 could promote HTNV entry via mediating membrane fusion between the late endosome membrane and the viral envelope membrane to release viral particles into the cytoplasm similar to the role of NPC1 in EBOV entry. To test this possibility, we relocated NPC1 to the cell membrane by replacing the transmembrane domain of wild-type NPC1 from ACE2 transmembrane domain and constructed a previously reported cell-membrane-localized NPC1 truncation containing only the MLD domain and a transmembrane domain (**Figure 3D**; **Supplementary Figure 1B**). The wild-type NPC1 or two modified NPC1 constructs which overexpressed 293T cells still held the same accessibility to HTNV particles than normal 293T cells (**Figure 3E**) and similar fusion efficacy between the HTNV envelope membrane and the cell membrane (**Figure 3F**; **Supplementary Figure 2A**). Taken together, we conclude that NPC1 is not implicated in the HTNV entry process.

### NPC1 regulates cellular cholesterol distribution to favor HTNV infection

**Figure 4:**
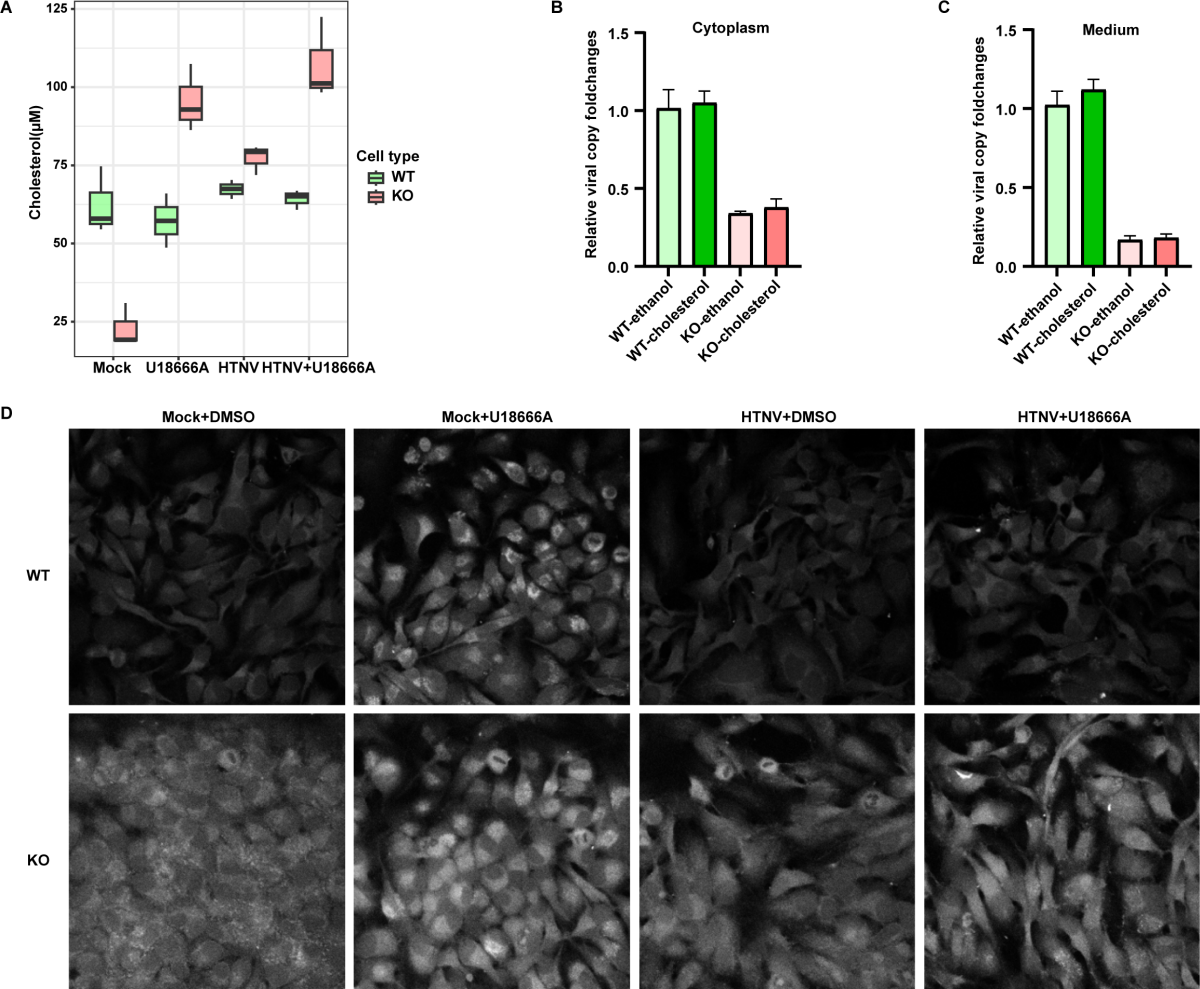
NPC1 disrupts cellular cholesterol distribution. **(A)** Wild-type and NPC1 knockout HeLa cells were treated with U18666A (10 µM) and HTNV (MOI = 10), cellular cholesterol levels were measured at 48 h post-infection. Non-treatment, U18666A treatment only or HTNV infection only were all parallel control. **(B)** Wild-type and NPC1 knockout HeLa cells were treated with cholesterol (10 µM) and HTNV (MOI = 1) or not, and viral genome copies within medium and cells of each group were quantified using qPCR. **(C)** The cellular cholesterol distribution of each group was visualized by Filipin staining following the same treatment as **(A)**. **(D)** The represented captures of Filipin staining for each group. WT: wild type HeLa cells, KO: NPC1 knockout HeLa cells. Mock+DMSO: cells were treated with the same amount of DMSO and without HTNV infection; Mock+U18666A: cells were treated with10 μM U18666A and without HTNV infection; HTNV+DMSO: cells treated with the same amount of DMSO and with HTNV infection for 48 hours (MOI=1); HTNV+U18666A: cells were treated with10 μM U18666A andwith HTNV infection for 48 hours (MOI=1).

NPC1, the core factor that transports cholesterol from the late endosome to the cytoplasm, essentially sustains cellular cholesterol homeostasis. As a result of the U18666A pretreatment for 12 h, HTNV replication was repressed (**Figure 2A**) but not with the 2-h pretreatment. As per the evidence provided in **Figure 3**, we turn to the effects of cellular cholesterol on HTNV infection. Firstly, we measured the cellular cholesterol level, and the results showed that (1) in wild-type HeLa cells, HTNV infection slightly increased the cellular cholesterol level, while the U18666A treatment slightly decreased the cellular cholesterol level (**Figure 4A**), (2) in NPC1 knockout HeLa cells, the cellular cholesterol level was significantly lower than that of wild-type HeLa cells, but either HTNV infection or U18666A treatment elevated the cellular cholesterol level (**Figure 4A**). These results suggest that HTNV infection regulating the cellular cholesterol level requires NPC1. To evaluate the roles of cholesterol deprivation observed in NPC1 knockout cells, we added external cholesterol into the medium to provide sufficient cholesterol. Exogen cholesterol supply did not affect HTNV replication in wild-type and NPC1 knockout HeLa cells (**Figures 4B, C**).

NPC1 depletion usually disrupts late endosome cholesterol efflux and the consequent endosomal cholesterol accumulation. We therefore detected cholesterol accumulation by Filipin staining. In wild-type cells, the U18666A treatment expectedly accumulated cholesterol within the late endosome, shown as a bright patch, while HTNV infection did not show any effect on cholesterol distribution pattern compared to the mock group (**Figure 4D**; **Supplementary Figure 2B**). Intriguingly, HTNV infection weakened the U18666A-treatment-induced endosomal cholesterol accumulation to a large degree (**Figure 4D**; **Supplementary Figure 2B**). In NPC1 knockout cells, cholesterol extensively condensed as the little puncta, while the U18666A treatment further converged cholesterol as the bigger patch (**Figure 4D**; **Supplementary Figure 2B**). The divergent cholesterol distribution pattern between NPC1 knockout cells and U18666A-treated cells indicated that the biological consequences following NPC1 depletion were more than cholesterol transport inhibition. Of note, HTNV infection failed to alleviate cholesterol accumulation without NPC1 (**Figure 4D**; **Supplementary Figure 2B**), which suggests that NPC1 is required to prevent abnormal endosomal cholesterol accumulation following HTNV infection. Taken together, NPC1 could manipulate cellular cholesterol distribution to promote HTNV replication.

### NPC1 mitigates innate immune response to promote HTNV infection

**Figure 5:**
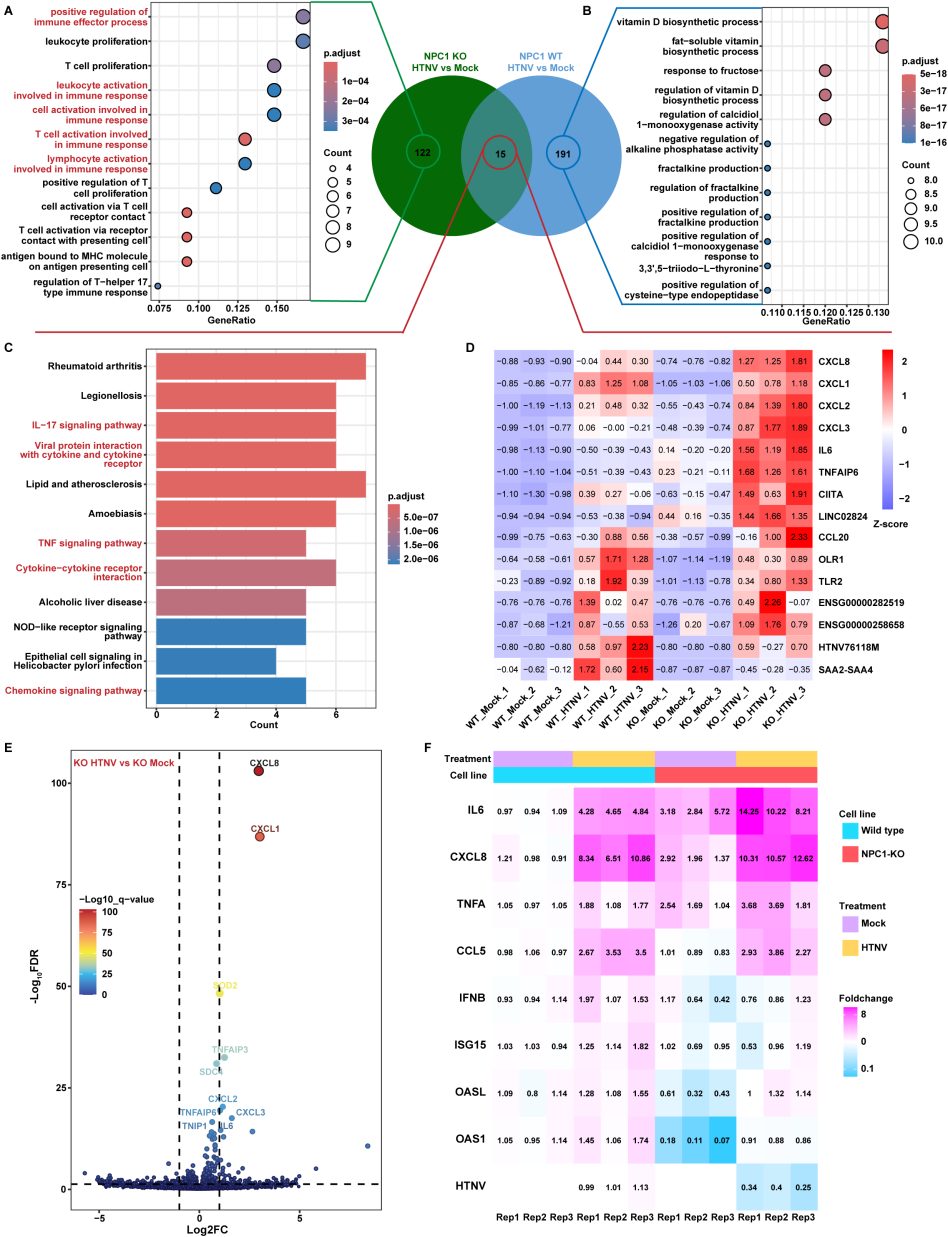
NPC1 mitigates innate immune response following HTNV infection. The significantly upregulated genes induced by HTNV infected among wild-type and NPC1 knockout HeLa cell lines were compared with each other. **(A, B)** GO term enrichment based on the genes unique to the NPC1 knockout cells **(A)** or wild-type cells **(B)**. **(C)** KEGG pathway enrichment based on the 15 overlapped genes. **(D)** FPKM-adjusted transcription levels of the 15 overlapped genes shown as heatmap (normalized by Z-score). **(E)** Differentially expressed genes between HTNV-infected cells and mock infected cells from NPC1 knockout cell line were depicted as scatter plot. **(F)** Wild-type and NPC1 knockout cells were infected with HTNV for 48 h or not, and the relative gene expressions of IL6, CXCL8, TNFA, CCL5, IFNB, ISG15, OASL, OAS1, and HTNV were quantified using qPCR.

To dissect the mechanism by which NPC1 favors HTNV infection, bulk RNA-seq has been performed following HTNV infection in wild-type and NPC1 knockout cells. NPC1 knockout triggered transcriptional change in more than 2,000 genes (**Supplementary Figure 3A**); however, when NPC1 knockout cells infected by HTNV were compared to wild-type cells infected by HTNV, the changes were too similar with NPC1 knockout itself to highlight HTNV-infection-specific gene expression changes (**Supplementary Figure 3B**). Consequently, we compared the differentially expressed genes (DEGs) induced by HTNV infection with NPC1 knockout cells and wild-type cells to specifically capture the genes changed by NPC1 knockout following HTNV infection (**Figures 5A, B**). The number of downregulated genes was too small to have biological significance, while 122 out of 138 upregulated genes were unique to the NPC1 knockout group (**Figure 5A**) and 191 out of 206 upregulated genes were unique to the wild-type group (**Figure 5B**). This strongly supported that NPC1 totally altered the host transcription response to HTNV infection. Specifically, the 191 upregulated DEGs unique to the wild-type group were mainly involved in vitamin D biosynthesis (**Figure 5B**), while the 122 upregulated DEGs unique to the NPC1 knockout group were mostly enriched in immune response pathways (**Figure 5A**), which suggested that NPC1 knockout indeed amplified the innate immune response following HTNV infection. Consistently, the 15 overlapping genes were highly enriched in the innate immune response involving IL-17, TNF, and cytokine production pathways (**Figure 5C**), which were induced by HTNV infection and had further elevated transcription levels in the NPC1 knockout group (**Figure 5D**). In addition, NPC1 knockout not only strengthened the expression of genes in the IL-17 pathway but also activated a broader gene transcription within the IL-17 pathway, which was supported by KEGG enrichment results based on the 122 upregulated DEGs unique to the NPC1 knockout group (**Figure 5E**; **Supplementary Figure 3C**). In detail, HTNV infection extensively activated the gene expression of all four branches in the IL-17 pathway in NPC1 knockout cells (**Supplementary Figure 3D**). Finally, we tested the transcription changes of some representative genes by qRT-PCR and found its gene expression pattern to greatly match the transcription changes observed in the RNA-seq data (**Figure 5F**). In addition, NF-κB, but not IRF3, was increased and specifically activated by NPC1 knockout (**Supplementary Figure 2C**), which further supports the observation found in RNA-seq data.

In summary, NPC1 knockout disrupted the cellular cholesterol distribution and amplified the host innate immune response post-HTNV infection. We concluded that NPC1-manipulated cellular cholesterol homeostasis might contribute to innate immune response mitigation and thus benefit HTNV replication, which is beyond the documented functions of NPC1 in the viral life cycle.

## Discussion

**Figure 6:**
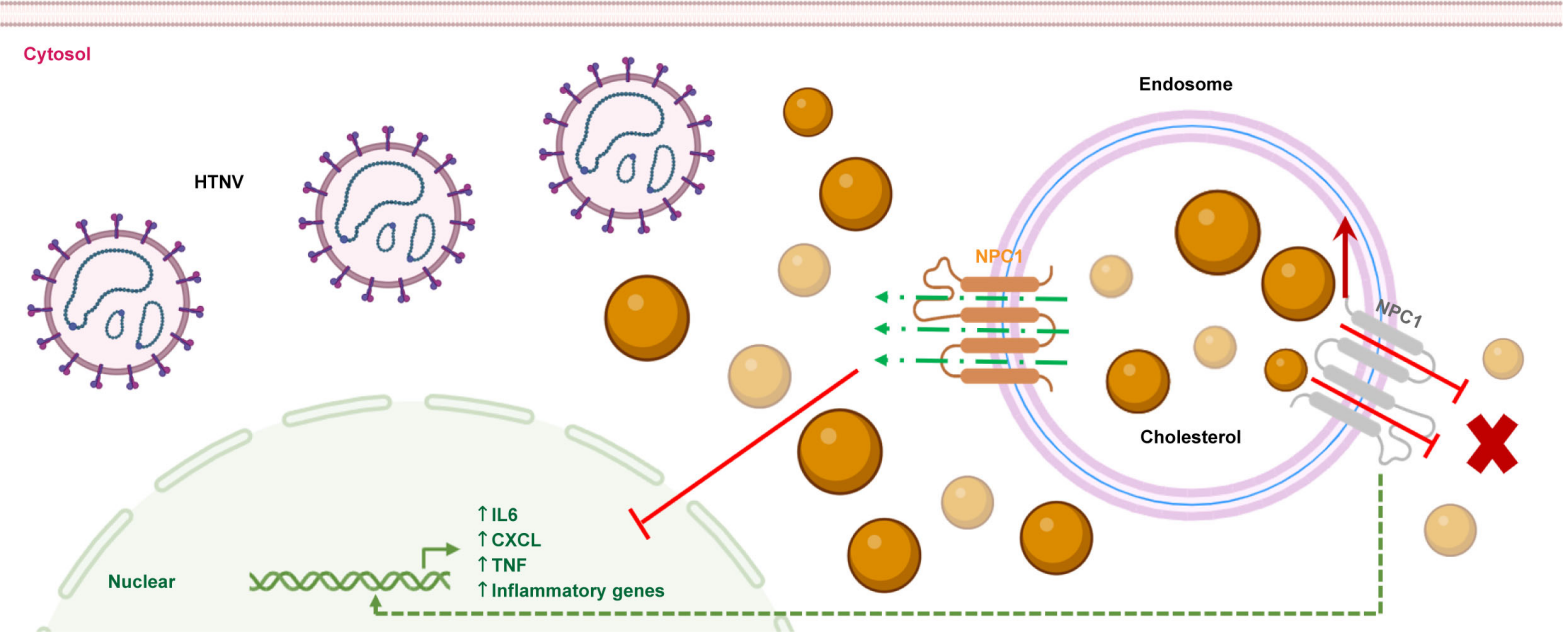
Diagram of NPC1 functioning in HTNV life cycle. HTNV infection triggers cellular cholesterol redistribution, which requires NPC1. Cholesterol extensively accumulated in late-endosome, thereby amplifying the innate immune response, when HTNV infected NPC1 defective cells. Consequently, NPC1 avoids abnormal endosomal cholesterol accumulation to mitigate innate immune response and promote HTNV replication.

Hantaan virus (HTNV) infection represents a persistent public health threat in Asia, yet the host determinants governing its replication and pathogenesis remain poorly understood. In this study, we identified NPC1 as a previously unrecognized host factor interacting with HTNV GP and uncovered a noncanonical function of NPC1 in modulating innate immune responses during HTNV infection (**Figure 6**). These findings not only broaden the functional spectrum of NPC1 in viral infection but also provide mechanistic insights into how HTNV exploits host lipid homeostasis to facilitate its replication.

The MaSIF virtual screening and IP-MS results converged on NPC1, together with structural modeling and biochemical validation, and this strongly supports the biological relevance of the NPC1–GP interaction. Interestingly, the predicted binding interface on NPC1 coincides with the domain that binds NPC2 or EBOV GP (49), indicating a potential conserved NPC1 recognition model shared between distinct viral families. Of note, the virtual screening criteria was a double-edged sword, such that stricter or looser constraints will result in missed potential candidates and high technical noise, which limit the reliability of virtual screening results presently. Our further results showed that NPC1 promotes HTNV replication by maintaining cellular cholesterol distribution. Both pharmacological inhibition and genetic deletion of NPC1 impaired HTNV replication, and cholesterol supplementation failed to rescue infection, indicating that global cholesterol availability is unlikely the primary limiting factor. Our unpublished data showed that pre-treated HUVECs with U18666A also repressed HTNV replication, which supports the conserved role of NPC1-mediated cholesterol redistribution in HTNV infection and suggests that it is not a cell-type-specific effect. Furthermore, the distinct cholesterol distribution pattern shown between NPC1-deficient cells and U18666A-treated cells suggests that NPC1 loss triggers broader perturbations in endosomal lipid organization.

Transcriptomic profiling further demonstrated that NPC1 deletion markedly amplifies innate immune responses following HTNV infection. Importantly, HTNV-induced gene expression changes were almost fully remodeled in the absence of NPC1, indicating that NPC1 acts as a central regulator controlling the magnitude of host transcriptional responses to infection. These results highlight a previously unappreciated immunomodulatory role of NPC1, extending beyond its established function in cholesterol transport. The disrupted cholesterol distribution in NPC1-deficient cells may perturb endosomal signaling hubs, thereby potentiating the activation of pattern-recognition receptor pathways during HTNV infection.

Our findings altogether reveal that NPC1 re-shaped the intracellular cholesterol landscape and dampened antiviral innate immune signaling, thereby contributing to HTNV replication. This mechanism represents a distinct departure from the well-documented role of NPC1 as an entry receptor for filoviruses and underscores the functional versatility of NPC1 in virus–host interactions. Moreover, the ability of HTNV to functionally exploit NPC1 for immune evasion suggests that targeting NPC1-mediated cholesterol trafficking or its downstream signaling effects may offer a promising therapeutic strategy. Given the involvement of NPC1 in the life cycles of multiple unrelated viruses, our study further supports the notion that NPC1 represents a potential antiviral target.

## Materials and methods

### Cells and virus

Vero E6 (ATCC: CRL-1587) cell line was obtained from the American Type Culture Collection. HeLa (KCB 86019YJ) and 293T (KCB 200744YJ) cell lines sensitive to HTNV infection were obtained from the Conservation Genetics Academy of Science (CAS) Kunming Cell Bank. Cells were grown in Dulbecco’s modified Eagle’s medium (DMEM, Gibco) supplemented with 10% fetal bovine serum (FBS, Gibco), penicillin (100 U/mL), and streptomycin (100 μg/mL) at 37 °C with 5% CO_2_ (52, 53). Hantaan virus (HTNV) 76–118 strain (GCA_000856085.1) was grown and titrated in Vero E6 cells as described previously (53). All cell infections with HTNV were conducted at Biosafety Level 2 (BSL-2) facility at Kunming Institute of Zoology (KIZ), Chinese Academy of Sciences (CAS).

### MaSIF virtual screen

All screening steps followed the instructions in the MaSIF toolkits (24, 26). HTNV GP crystal structure (7NKS) (48), which was used for target surface fingerprint extraction using the MaSIF-site module, was downloaded from RCSB Protein Data Bank. Meanwhile, 2,965 membrane protein structures were downloaded from AlphaFold Protein Structure Database and followed using MaSIF-site prediction and MaSIF-search descriptors. Finally, the MaSIF-seed program was run to screen the membrane proteins which may interact with HTNV GP protein potentially.

### Immunoprecipitation and mass spectrometry

For NPC1 immunoprecipitation coupled with HA or S-tag western blotting, 293T cells were transfected with a Flag-tagged NPC1 expression plasmid or an HTNV full-length GP expression plasmid tagged with an N-terminal HA and C-terminal S-tag for 24 h. After that, standard immunoprecipitation coupled with western botting steps was done as described previously (52). For immunoprecipitation coupled with mass spectrum (IP-MS) assay, cells were transfected with Flag-tagged HTNV Gn- and Gc-expressing plasmids for 24 h, and then cell lysates were harvested and Flag-specific immunoprecipitation steps followed the standard immunoprecipitation steps (52). The immunoprecipitated proteins were sent to the public instrument center of Kunming Institute of Zoology, Chinese Academy of Sciences, for liquid chromatography–mass spectrometry (LC–MS) analysis. The IP-MS results are available in **Supplementary Table 2**.

### Western blotting

Protein concentration was determined using Enhanced BCA Protein Assay Kit (Beyotime), and 30 μg of protein was loaded per well (52). The following antibodies were used: NPC1 (Abcam, 1:1,000), HA (Abcam, 1:2,000), S-tag (Abmart, 1:1,000), FLAG (Abcam, 1:2,000), β-tubulin (Abcam, 1:2,000), vinculin (CST, 1:1,000), HRP-labeled goat anti-mouse IgG (H+L) antibody (Invitrogen, 1:3,000), and HRP-labeled goat anti-rabbit IgG (H+L) antibody (Invitrogen, 1:3,000). The signal was detected after incubation with the Immobilon Western Chemiluminescent HRP Substrate (Millipore).

### Protein–protein docking and visualization

Eight proteins were predicted to bind the HTNV GP protein following the MaSIF virtual screening workflow, and protein complexes were docked by AlphaFold3 web service (https://alphafoldserver.com/) (15). All complexes colored by plDDT score were automatically outputted by AlphaFold3 web service, and the detailed HTNV GP–NPC1 complex and the zoomed-in interaction interface were all visualized using ChimeraX.

### NPC1 knockdown, knockout, and rescue

NPC1 target shRNA and sgRNA sequences were synthesized and cloned into pLKO.1 and lentiCRISPR V2 backbone, respectively, by Beijing Tsingke Biotech (the shRNA and sgRNA sequences are provided in **Supplementary Table 1**), while Flag-tagged NPC1 CDS was cloned into pTomo backbone. The lenti-viruses of interest were rescued by co-transfection with psPAX2, pMD2.G, and target plasmid into 293T cells. The viral titer was determined by TCID_50_, and shRNA or NPC1 carrying lenti-virus was transduced into wild-type HeLa cells or NPC1 knockout cells with polybrene transfection reagent (10 µg/mL) at multiplicity of infection (MOI) = 10, while sgRNA carrying lenti-virus was transduced into wild-type HeLa cells at MOI = 0.1. All shRNA- and sgRNA-transduced cells were incubated with 5 µg/mL puromycin to get the successfully transduced cells and cell clones. The surviving NPC1 knockdown cell populations or NPC1 knockout cell clones were used for knockdown or knockout efficiency test and further experiments. The NPC1-transduced cells were sorted by flow cytometry (Beckman Moflo Asrios EQs) to obtain NPC1-rescued HeLa cells.

### Attachment and penetration detection

Wild-type, NPC1 knockout, and NPC1-rescued HeLa cells were grown to confluence in a 12-well plate. HTNV was inoculated into cells at MOI = 10 after three washes with ice-cold PBS, and the cells were incubated at 4°C for 2 h. Then, the medium was discarded, and the cells were collected as virus attachment sample following three washes with ice-cold PBS. Meanwhile, the remaining cells with the same treatment as mentioned above were returned to 37°C for 2 h. After that, the medium was discarded, and the cells were collected as virus penetration sample following 1 min of incubation with 0.25% trypsin and three times of rinsing with ice-cold PBS. 293T cells were grown on a 24-well plate, the cells were infected with HTNV at an MOI = 10 following transfection with NPC1 constructs (1 µg per well) for 24 h, and the attachment sampling was the same as the steps mentioned above. Total RNA was extracted using Trizol, and viral RNA copies were quantified by qRT-PCR (53).

### Plasmid construction

All of the plasmids used in our study were constructed through *in vitro* homologous recombination based on ClonExpress II One Step Cloning Kit (Vazyme). Full-length HTNV GP CDS flanked with HA and S-tag at the N- and C-terminal was inserted into the pCAGGS backbone, while the N-terminal Flag-tagged HTNV Gn (1-601aa) and Gc (643-1135aa) coding sequences were inserted into the pCS2 backbone. The C-terminal Flag-tagged full-length NPC1 and two modified NPC1 sequences were inserted into the pTomo backbone, and the detailed sequence component is shown in **Supplementary Figure 1B**. pCMV-GFP1–10 and pCMV-16×GFP11 were constructed by Beijing Tsingke Biotech. All primers involved are provided in **Supplementary Table 1**.

### Immunofluorescence

293T cells were plated on coverslips in a 24-well plate for 12 h, and then the cells were transfected with the NPC1 constructs for 24 h. Subsequently, the immunofluorescence-related steps followed the protocol described previously (52). The details about the antibodies are as follows: mouse anti-Flag (1:500) and goat anti-mouse conjugated to Alexa Fluor 488 (Invitrogen; 1:1,000). Images were captured using a Zeiss LSM880 confocal microscope (Zeiss).

### Antibody blockage assay

HeLa cells were grown in 6-cm dishes and detached using 2 mM EDTA, and the indicated NPC1 antibody (0, 125, 250, 500, 1,000, and 2,000 ng/mL) was incubated with the cells at room temperature for 1 h. Then, 10^5^ HeLa cells were incubated with 10^6^ TCID_50_ HTNV for 2 h at 4°C. After that, the cells were stained with anti-NP polyclonal antibody for 2 h at 4°C and then with goat anti-ribbit IgG(H+L) Alexa Fluor 594 for 1 h at 4°C, which was interspersed with three washes with ice-cold PBS. A total of 10^4^ cells were analyzed using a BD LSR Fortessa Flow Cytometer (BD Bioscience), and the data were analyzed using the FlowJo software version 10 (TreeStar).

### Membrane fusion assay

293T cells were grown on a 24-well plate for 12 h, and then these were transfected with NPC1 constructs and GFP1–10 or HTNV GP and 16×GFP11 for 24 h. The cells were suspended by pipetting following 30 s of trypsin incubation. Subsequently, HTNV GP- and NPC1 construct-transfected cells were mixed and plated in the same well of the 24-well plate, and GFP signals were captured using Primovert iLED microscopy (Zeiss) after 24 h of co-culture. ACE2-SARS-CoV2 S and empty vector–empty vector were the parallel positive control and negative control, respectively.

### Cellular cholesterol level detection

Wild-type and NPC1 knockout HeLa cells were grown on a six-well plate for 12 h, 10 µM U18666A and HTNV (MOI = 1) were added to the cells, and the cellular cholesterol level was measured using Tissue Total Cholesterol (TC) Content Assay Kit (APPLYGEN) at 48 h post-infection. Non-treatment (DMSO and DMEM), U18666A treatment only, or HTNV infection only were the parallel controls.

### Filipin staining

Wild-type and NPC1 knockout HeLa cells were plated on coverslips in a 24-well plate for 12 h; 10 µM U18666A and HTNV (MOI = 10) were added to the cells. At 48 h post-infection, the cells were fixed with 4% paraformaldehyde and permeabilized using 0.5% Triton X-100 in PBS buffer for 10 min. Then, the cells were stained overnight using 0.05 mg/mL Filipin complex, and images were captured using a Zeiss LSM880 confocal microscope (Zeiss). Non-treatment (DMSO and DMEM), U18666A treatment only, or HTNV infection only were the parallel controls.

### Chemical treatments

U18666A (10 µM) was pre-incubated with HeLa cells for 12 h (if there was no separate statement), then HTNV was inoculated, and U18666A was presented in the medium until the indicated sampling time point. Ethanol-dissolved cholesterol (20 µM) and HTNV (MOI = 1) were added to the medium simultaneously, and sampling was done at 48 h post-infection (cholesterol was presented in the medium until sampling).

### Isolation of viral genomic RNA for qRT-PCR analyses

HeLa cells were infected with HTNV following the indicated treatment, and then the medium and cells were harvested based on the experiment design. Viral RNA in the medium was extracted using nucleic acid extraction instruments VNP-32P (Vazyme) with Virus DNA/RNA Extraction Kit 2.0 (Vazyme), and viral quantity was measured using HiScript II One Step qRT-PCR Probe Kit (Vazyme) in triplicate. RNAiso Plus (TaKaRa) was used for cellular RNA purification according to the manufacturer’s instruction, and 1 µg RNA was reverse-transcribed using Prime ScriptRT Reagent Kit with gDNA Eraser (TaKaRa) and stored at -20°C. Real-time PCR runs in triplicate with 50 ng cDNA T5 Fast qPCR Mix (Probe) (TSINGKE). The sequences of the primers and probes used are provided in **Supplementary Table 1**. Relative gene expression or viral genome RNA fold changes were quantified using the ΔΔCt method. To determine the relative RNA content, the average Ct values for each sample were subtracted from the average Ct values of 18s RNA to get the ΔCt value, and the ΔΔCt value was obtained by subtracting the mean ΔCt value of the control group from that of the experimental group: ΔΔCt = (Ct_experimental_ − Ct_experimental_ 18s) − (Ct_control_−Ct_control_ 18s). The fold change value is 2^−ΔΔCt^.

### Bulk RNA sequencing

Wild-type and NPC1 knockout HeLa cells were seeded in six-well plates at 5 × 10^5^ cells per well and cultured overnight culture, and HTNV was inoculated into the cells at MOI = 10. Vero E6 cell culture medium inoculation was the mock infection. We have four groups, including WT-Mock, WT-HTNV, KO-Mock, and KO-HTNV, with three independent replicates each. All of the samples were collected at 48 h post-infection by adding Trizol to lyse cells (54). The total RNA was extracted, and the poly(A)-enriched RNA was used for library construction and sequencing on the Illumina NovaSeq XPlus (Novogene).

### Bioinformatics

The following analysis pipeline was used and described previously (54). In detail, raw data were trimmed using trim_galore (v0.6.7) with pair-end reads model and then aligned to the combined genome containing human hg38.p14 (downloaded from the GENCODE) and viral HTNV 76-118 (GCA_000856085.1, downloaded from NCBI) by hisat2 (v2.2.1) (55). SAM file was converted to BAM file and indexed using SAMtools (v1.16.1) (56), and gene counts were quantified using featureCount (v2.0.1) (57) from the transcript level. All further analyses and figure generation were accomplished in R (v4.3.2). Specifically, differential analysis was performed using DESeq2 (58); KEGG and GO enrichment, respectively, was completed using clusterProfiler (59) and plotted using pathview and GOplot. The heatmap was created using ggplot2.

### Quantification and statistical analysis

Statistical tests are indicated in the figure legends. Statistical significance is denoted in the figures using the following notations: ns, not statistically significant; ****p < 0.0001; ***p < 0.001; **p < 0.01; *p < 0.05. All error bars represent standard deviation (SD). All statistical analyses were performed in R4.3.2.

## Data Availability

The datasets presented in this study can be found in online repositories. The names of the repository/repositories and accession number(s) can be found in the article/**Supplementary Material**.

## Supplementary material

Supplementary Figure 1 NPC1 is predicted to interact with HTNV GP. **(A)** All of the candidates outputted by MaSIF screening were docked with HTNV GP by AlphaFold3, and the visualized protein complexes were colored by plDDT (predicted Local Distance Difference Test, the confidence of prediction accuracy of each residue) score. **(B)** Diagram depicting the sequence component of each NPC1 construct.

Supplementary Figure 2 NPC1 regulates cellular cholesterol distribution. **(A, B)** Fluorescence intensity quantification based on three independent captures of each group corresponding to **Figure 3F** **(A)** and **Figure 4D** **(B)**. **(C)** Wild-type and NPC1-KO HeLa cells were infected with HTNV (MOI = 1) or mock-infection for 48 h; then, the cell lysate was used for pNF-κB, NF-κB, pIRF3, IRF3, NPC1, NP, and GAPDH protein level measurement by western blotting.

Supplementary Figure 3 NPC1 regulates innate immune response following HTNV infection. **(A, B)** Differentially expressed genes between NPC1 knockout cells and wild-type cells under mock infection **(A)** and HTNV infection **(B)** context, shown as scatter plot. **(C)** KEGG pathway enrichment of the 122 genes unique to NPC1 knockout group (the same genes in **Figure 5A**). **(D)** Overview of gene expression changes between HTNV-infected cells and mock-infected cells within IL-17 pathway when NPC1 was knockout.
