# Supplementary figures and images for: NPC1 promotes HTNV replication by controlling innate immune response
Source: Front Immunol. 2026 Jun 5;17:1811629. doi: 10.3389/fimmu.2026.1811629 (PMC13279697; doi:10.3389/fimmu.2026.1811629)

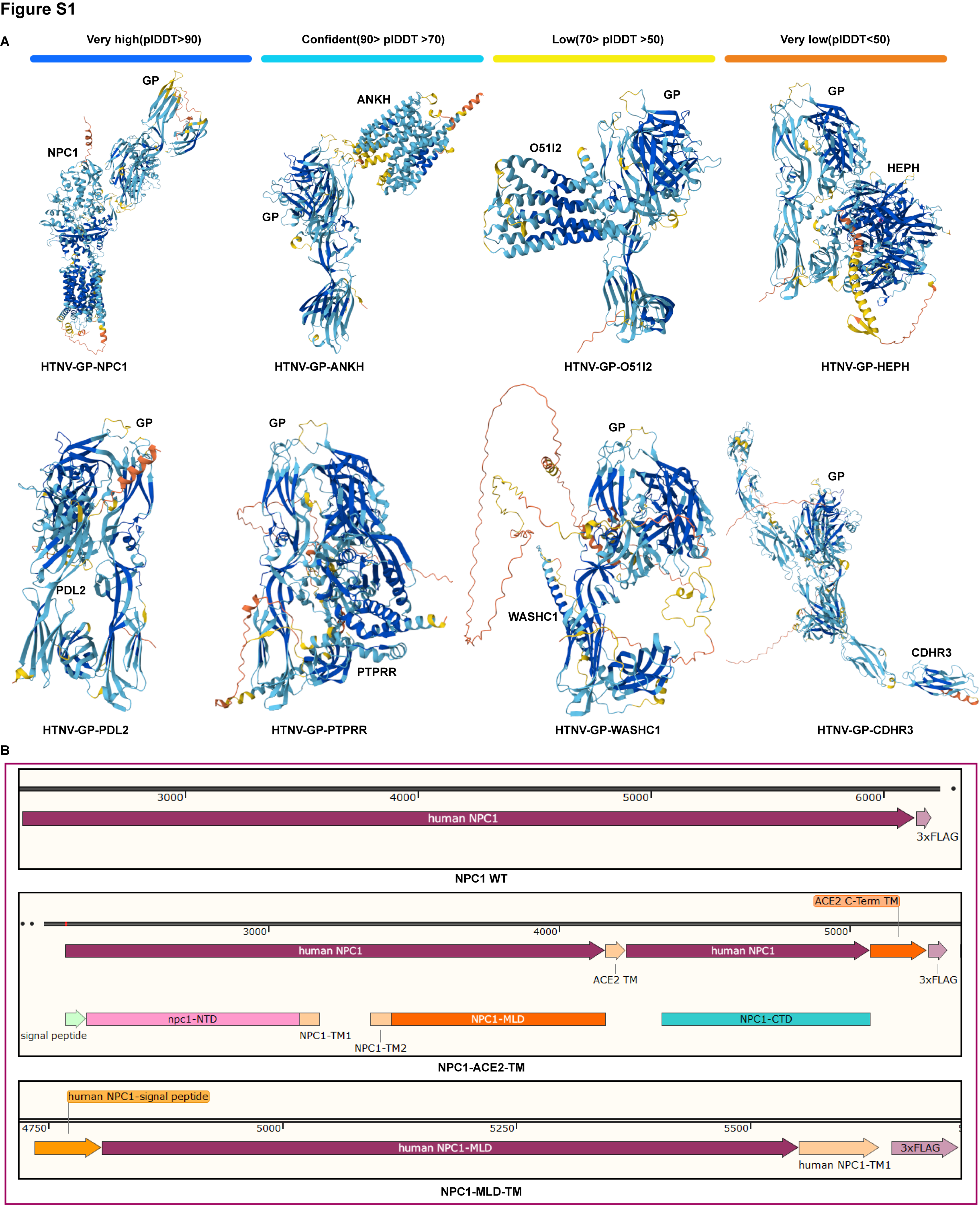

Supplement: Supplementary Figure 1 — NPC1 is predicted to interact with HTNV GP. (A) All of the candidates outputted by MaSIF screening were docked with HTNV GP by AlphaFold3, and the visualized protein complexes were colored by plDDT (predicted Local Distance Difference Test, the confidence of prediction accuracy of each residue) score. (B) Diagram depicting the sequence component of each NPC1 construct. [file Image1.tif]

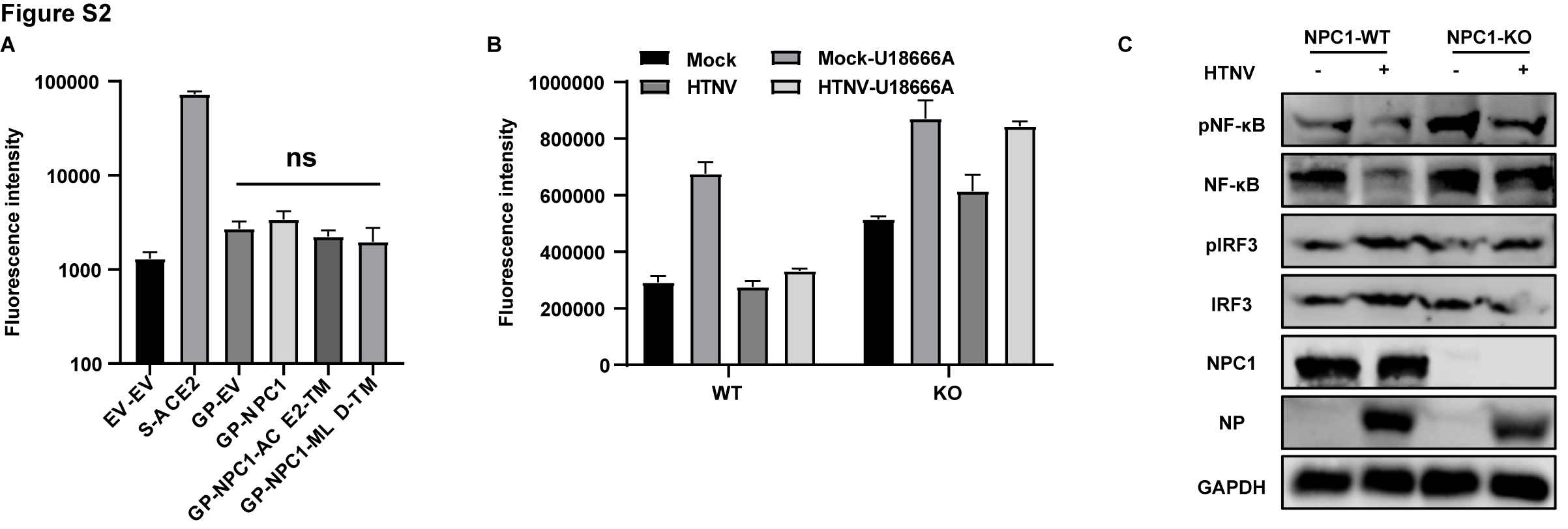

Supplement: Supplementary Figure 2 — NPC1 regulates cellular cholesterol distribution. (A, B) Fluorescence intensity quantification based on three independent captures of each group corresponding to Figure 3F (A) and Figure 4D (B). (C) Wild-type and NPC1-KO HeLa cells were infected with HTNV (MOI = 1) or mock-infection for 48 h; then, the cell lysate was used for pNF-κB, NF-κB, pIRF3, IRF3, NPC1, NP, and GAPDH protein level measurement by western blotting. [file Image2.tif]

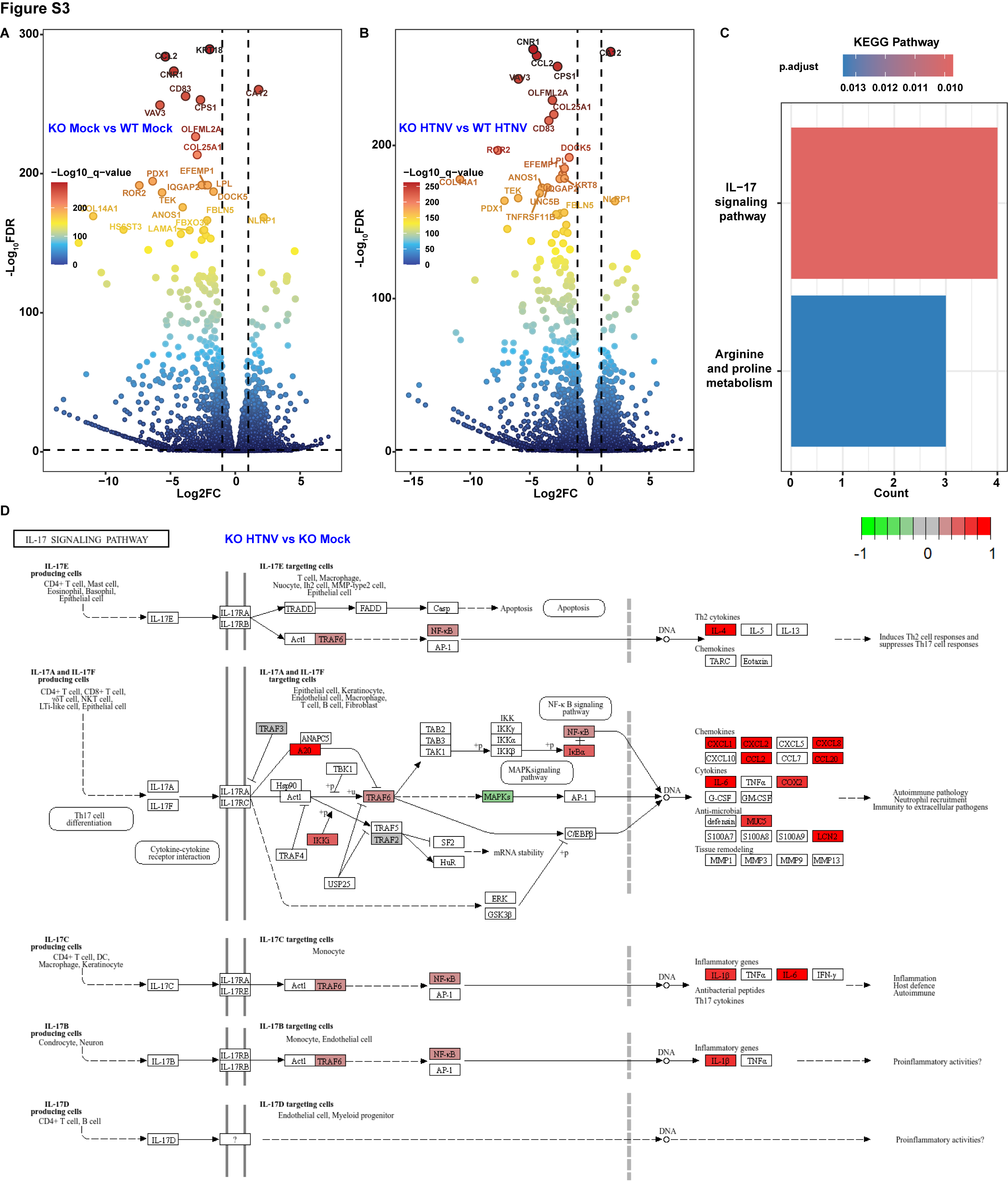

Supplement: Supplementary Figure 3 — NPC1 regulates innate immune response following HTNV infection. (A, B) Differentially expressed genes between NPC1 knockout cells and wild-type cells under mock infection (A) and HTNV infection (B) context, shown as scatter plot. (C) KEGG pathway enrichment of the 122 genes unique to NPC1 knockout group (the same genes in Figure 5A). (D) Overview of gene expression changes between HTNV-infected cells and mock-infected cells within IL-17 pathway when NPC1 was knockout. [file Image3.tif]
