# Supplementary material for: NPC1 promotes HTNV replication by controlling innate immune response
Source: Front Immunol. 2026 Jun 5;17:1811629. doi: 10.3389/fimmu.2026.1811629 (PMC13279697; doi:10.3389/fimmu.2026.1811629)
Supplement: Supplementary file 4 [file Table1.docx]

**Table S1. Oligonucleotides used in this study, related to Materials and methods.**

| Plasmid construction | |
| --- | --- |
| Tomo-HA-Vector-F | CTCGAGGGTGGATCATACC |
| Tomo-HA-Vector-R | GGATCCACCAGCGTAATCTG |
| Tomo-M-ORF-F | CAGATTACGCTGGTGGATCCATGGGGATATGGAAGTGG |
| Tomo-M-ORF-R | GGTATGATCCACCCTCGAGTGATTTTTTATGCTTCCTTACGG |
| NPC1-NTD-F | GATTCTAGAGCTAGCGAATTCGCCACCATGGGCGGATCCTGTGTTTGGTATGGAGAGTG |
| NPC1-MLD-R | AAACAATCAGCCATATACTGTCACTTTCACGATTTAGTTCATCTTCAATACTTCG |
| ACE2-TM-F | CAGTATATGGCTGATTGTTTTTGGAGTTGTGATGGGAGTGATAGTGGTTGGCATTGTCA |
| ACE2-TM-R | CATGGTCTTTGTAGTCCTCGAGCCCAGTGAAGATCAGGATGACAATGCCAACCACTATC |
| NPC1-CTD-F | GTCATCCTGATCTTCACTGGGAGCTGTTTGTTTCGCTTCTTC |
| NPC1-CTD-R | GATGGTCAGGTACTGTTCGTAGAAGACATAAAACACACTGTAAGGA |
| ACE2-C-Term-F | ACGAACAGTACCTGACCATCATATGGCTGATTGTTTTTGGAGT |
| ACE2-C-Term-R | CATGGTCTTTGTAGTCCTCGAGAAAGGAGGTCTGAACATCAT |
| pCAGGS-Gp-F | ATGGGGATATGGAAGTGGCT |
| pCAGGS-EcoR1-R | GAATTCTTTGCCAAAATGATGAGACAG |
| pCAGGS-HA-F | ATCATTTTGGCAAAGAATTCGCCACCATGGGCGGAAGTTACCCATACGACGTACCAG |
| pCAGGS-HA-R | AGCCACTTCCATATCCCCATGGATCCGCCAGCGTAATCTGGTACGTCGTATGGGTAA |
| qPCR primers | |
| 18S-F | GTAACCCGTTGAACCCCATT |
| 18S-R | CCATCCAATCGGTAGTAGCG |
| S(+)-F | CTGGCTGAGCATCATCGTCT |
| S(+)-R | AAATCGAATCCGGGTCCCTT |
| S-F | ACATCTGAGGAGAAGCTACGG |
| S-R | GGCAACCATGAAGAGCACAA |
| S-probe | FAM-AGCATCATCGTCTATCTTACATCC |
| NPC1-F | CTCCTGCTGCTACTGTGTCC |
| NPC1-R | CAATGGTTTTGGTGGGCCAG |
| IL6-F | ACTGGTCTTTTGGAGTTTGAGGT |
| IL6-R | GCATCTAGATTCTTTGCCTTTTTCT |
| CXCL8-F | CACTGCGCCAACACAGAAAT |
| CXCL8-R | TGAATTCTCAGCCCTCTTCAAA |
| TNFα-F | GCCGCATCGCCGTCTCCTAC |
| TNFα-R | CCTCAGCCCCCTCTGGGGTC |
| CCL5-F | ACCCAGCAGTCGTCTTTGTC |
| CCL5-R | CAGGTTCAAGGACTCTCCATCC |
| IFNB1-F | TCTCCTGTTGTGCTTCTCCAC |
| IFNB1-R | GGCAGTATTCAAGCCTCCCA |
| ISG15-F | AGGAATAACAAGGGCCGCAG |
| ISG15-R | CCTCGAAGGTCAGCCAGAAC |
| OASL-F | CTGAGGCAGGAGCATTTCCA |
| OASL-R | CTCAGAAACGCCACCAGCTC |
| OAS1-F | AGCTTCGTACTGAGTTCGCT |
| OAS1-R | AGCTTGACATAGATTTGGGGGT |
| shRNA and sgRNA sequences | |
| shNPC1-1 | GGTCACAACCAATCCAGTTGA |
| shNPC1-2 | GGTGCTGAAGATGGAACAAGC |
| sgNPC1-1 | AAAGAGTTACAATACTACGT |
| sgNPC1-2 | GTACAATGCCTGCCGGGATG |
| sgNPC1-3 | GAATTGCATATGGGGACAAG |
| sgNPC1-4 | GTTCATTACTGCGTGTTCGTC |
